# Genome Sequence of Atyrau-5BJN(IL18), a Recombinant Lumpy Skin Disease Virus with Knockout of Virulence Genes

**DOI:** 10.1128/mra.00380-22

**Published:** 2022-06-21

**Authors:** Aisha U. Issabek, Mukhit B. Orynbayev, Kulyaisan T. Sultankulova, Asylulan Amirgazin, Kunsulu D. Zakarya, Zamira Omarova, Olga V. Chervyakova

**Affiliations:** a Research Institute for Biological Safety Problems, Gvardeyskiy, Republic of Kazakhstan; b National Center for Biotechnology, Nur-Sultan, Republic of Kazakhstan; DOE Joint Genome Institute

## Abstract

Here, we present the coding sequence of the genome of the recombinant lumpy skin disease virus (LSDV) Atyrau-5BJN(IL18), obtained by knocking out four genes in the genome of a virulent field LSDV isolate. Genome sequencing confirmed the deletion of genes and the insertion of a foreign sequence in the viral genome.

## ANNOUNCEMENT

Lumpy skin disease virus (LSDV), genus *Capripoxvirus* of the family *Poxviridae*, is the causative agent of an infectious disease in cattle. Rapid diagnostics and vaccination are the basis for controlling the disease spread. The development of molecular biology methods has made it possible to obtain attenuated viruses by targeted mutagenesis of the viral genome (1).

Here, we present the coding sequence of the genome of the recombinant lumpy skin disease virus (LSDV) Atyrau-5BJN(IL18), obtained by knocking out four genes in the genome of a virulent field LSDV isolate. The primary virulent virus was isolated from an infected cow during an outbreak of lumpy skin disease in Kazakhstan in July 2016 (ProMED 20160722.4363497). Initially, four passages were performed in the MDBK cell line, and then four genes were knocked out by homologous recombination with transient dominant selection of recombinant viruses (2). We anticipate that the recombinant Atyrau-5BJN(IL18) will be harmless, immunogenic, and protect cattle during challenge with virulent virus (3).

The recombinant virus was produced in lamb testicle cell culture and purified by centrifugation in a sucrose density gradient (4). Genomic DNA was isolated using TRIzol reagent (Invitrogen, USA) according to the manufacturer’s protocol.

A DNA library was prepared using the Nextera XT DNA library preparation kit (FC-131-1024). Sequencing was performed on the Illumina MiSeq platform using the MiSeq reagent kit v3 (600 cycles; MS-102-3003). Quality control of the reads was carried out using FastQC v0.11.15 (5). Overall, 2,268,136 reads were obtained and trimmed to at least Q30 from the 5′ end on 19 nucleotides and from the 3′ end on 3 nucleotides using the programs Sickle v1.33 (6) and SeqTK v1.3-r106 (7).

*De novo* genome assembly was performed using the program SPAdes v3.13 (8) with a k-mer length of 127 and the “–careful” option. As a result, a contig with a length of 145,167 bp was obtained; it was identified using blastn and the nucleotide collection (nt) database (9) as an LSDV genome.

To determine the depth of sequencing, the reads were mapped to the corresponding assemblies using the BWA-mem v0.7.17 algorithm (standard settings) (10), and the depths for each nucleotide and the median were calculated using SAMtools v1.15.1 (11). The sequencing depth was 255×.

Inverted terminal repeat sequences were PCR amplified using AccuPrime *Taq* DNA polymerase, high fidelity, and determined by Sanger sequencing using the BigDye Terminator v3.1 cycle sequencing kit (Applied Biosystems, Austin, TX) on the 3130xl genetic analyzer (Applied Biosystems, Hitachi, Japan). The resulting contigs were manually combined into a sequence of 148,628 bp with an average G+C content of 25.87%, evenly distributed. About 600 bp were missing from both the 5′ and 3′ ends of the genome compared to the genome sequences present in GenBank.

The BLAST results showed that Atyrau-5BJN(IL18) has 100% identity with LSDV field isolates from Israel (2012; GenBank accession number KX894508), Turkey (2014; MN995838), Greece (2015; KY829023), Kazakhstan (2016; MN642592). The identity with the NCBI Reference Sequence NC_003027 was 99.88%. The query coverage in both cases was 99%.

Genome annotation was performed using GATU software relative to the LSDV isolate Kubash/KAZ/16 (GenBank accession number MN642592) (12). In addition to the targeted mutations introduced into the genome of the recombinant virus, three nucleotide substitutions were identified that would lead to amino acid substitutions (Table 1).

**TABLE 1:** Nucleotide modifications and their effects on the encoding sequences

Data for strain:				Nucleotide modification(s)	Change in coding sequence
Atyrau-5BJN(IL18)		Kubash/KAZ/16			
ORF	Position	ORF	Position		
rLSDV004	1807–1884	LD005	2298–2810	Deletion	1–149 aa deletion
rLSDV007	3773–3853	LD008	4688–5515	Deletion	20–266 aa deletion
rLSDV064	54985–56190	LD067	56645–57178	Insertion	Gene disrupted by insertion of bovine interleukin-18 coding sequence.
rLSDV141	132881–132999	LD143	133868–134272	Deletion	8–134 aa deletion
rLSDV142	133262, 133289, 133340	LD144	134532, 134559, 134610	Mutations	H75R, I84T, L101S

Genome sequencing confirmed the deletions of genes and the insertion of a foreign sequence in the viral genome (Table 1).

### Data availability.

The genome sequence of the lumpy skin disease virus Atyrau-5BJN(IL18) has been deposited at GenBank under accession number ON005067, and the raw data have been submitted to the SRA under BioProject accession number PRJNA825391.
